# Minimal renal toxicity after Rituximab DHAP with a modified cisplatin application scheme in patients with relapsed or refractory diffuse large B-cell lymphoma

**DOI:** 10.1186/s12885-016-2289-y

**Published:** 2016-04-11

**Authors:** K. Lisenko, F. McClanahan, T. Schöning, M. A. Schwarzbich, M. Cremer, T. Dittrich, A. D. Ho, M. Witzens-Harig

**Affiliations:** Department of Hematology, Oncology and Rheumatology, University Hospital Heidelberg, Im Neuenheimer Feld 410, Heidelberg, 69120 Germany; Centre for Haemato-Oncology/Barts Cancer Institute, Queen Mary University of London, London, UK; Pharmacy Department, University Hospital Heidelberg, Heidelberg, Germany

**Keywords:** R-DHAP, DLBCL, Renal chemo toxicity

## Abstract

**Background:**

Rituximab (R) in combination with DHAP is a widely accepted salvage regimen for patients with relapsed or refractory diffuse large B-cell lymphoma (DLBCL). A common adverse effect of this protocol is renal toxicity which may result in treatment discontinuation. Assuming that a lower single dose of cisplatin over several days would reduce renal toxicity, our institution has chosen to administer cisplatin in a dosage of 25 mg/m^2^ per day as a 3-h infusion over 4 consecutive days.

**Methods:**

In this study, we analyzed the renal function of 122 patients with relapsed or refractory DLBCL treated with R-DHAP at our institution. Overall, 256 R-DHAP cycles were administered. 31 (25 %), 61 (50 %), 14 (12 %) and 16 (13 %) patients received one, two, three or four R-DHAP courses, respectively.

**Results:**

A glomerular filtration rate (GFR) decrease was observed after each R-DHAP cycle. However, in none of the subgroups the median GFR was lower than 60 ml/min/1.73 m^2^. In most patients, only renal impairment stage I and II was observed. Renal impairment stage III was seen in 10 % and stage IV only in 1 % of patients.

**Conclusion:**

We conclude that a modified R-DHAP regimen with administration of cisplatin 25 mg/m^2^ over 4 consecutive cycles leads only to minimal renal toxicity.

## Background

The addition of the anti-CD20 monoclonal antibody rituximab (R) have significantly improved the outcome and survival of patients with diffuse large B-cell lymphoma (DLBCL), and complete responses (CRs) after first line therapy are observed in 75–80 % [1–3]. However, approximately one-third of patients develop relapsed or refractory disease [4]. For eligible patients, inductionchemoimmunotherapy, and in case of chemosensitive disease, high-dose (HD) chemotherapy and autologous stem cell transplantation (ASCT) are the current standard of care [5]. Such salvage therapies typically consist of cytotoxic agents that have not been used in first line therapy. One established regime in relapsed or refractory DLBCL is R-DHAP. R-DHAP combines rituximab (375 mg/m^2^), given intravenously (i.v.) one day before chemotherapy, cisplatin (100 mg/m^2^), typically administered i.v. by continuous infusion over 24 h, followed on day 2 by cytarabine (2 g/m^2^) in a 3-h long infusion (repeated after 12 h), and oral dexamethasone (40 mg/d for 4 consecutive days) repeated after 21 days for 2–4 cycles. However, this regimen is associated with severe adverse effects that often require discontinuation of treatment, such as myelosuppression, infections and renal toxicity. For instance, in a phase II trial in relapsed non-Hodgkin lymphoma evaluating the toxicity and efficacy of adding 4 doses of rituximab to a conventional DHAP regimen grade III or IV nephrotoxicity was observed in 7 % of patients after two R-DHAP courses [6]. Moreover, in a randomized phase III trial in relapsed or refractory DLBCL comparing two salvage regimes, stage IV renal toxicity occurred in 6 % of patients treated with three cycles of conventional R-DHAP [7]. Because of the efficacy of the R-DHAP regimen, attempt to reduce nephrotoxicity e. g. by substitution of cisplatin with oxaliplatin has been successfully made [8]. Another attractive approach to limit renal toxicity is to spread the cisplatin application over several days. For example, cisplatin can be administered for 4 consecutive days as a 3 h infusion, consisting of 25 mg/m^2^ each. Moreover, the administration of cisplatin for several hours on four consecutive days, instead of a 24 h infusion, allows treating patients with this regimen on an outpatient basis. However, a systematic evaluation of the toxicity and efficacy of this modification is warranted. In the current study we therefore retrospectively analyzed 122 patients with relapsed or refractory DLBCL treated with the modified R-DHAP regimen at our institution. Our aims were to evaluate the efficacy and renal toxicity of this salvage therapy regimen.

## Methods

### Patient selection

All patients with relapsed or refractory DLBCL that were treated with R-DHAP at our institution from July 2002 to July 2013 received the modified R-DHAP regimen, under a hypothetical, but so far not verified, assumption that a lower single dose of cisplatin over several days would reduce renal toxicity. Overall, 122 patients were included into this analysis. All patients had histologically confirmed DLBCL. Clinical characteristics (age, gender, stage at diagnosis), previous therapy, time to relapse and serum creatinine before each individual and after the last R-DHAP course were collected and retrospectively analyzed. This analysis was approved by the ethics committee University Hospital Heidelberg without an informed consent of the patients with regard to its retrospectivity. Research was carried out in compliance with the Helsinki Declaration.

### Modified R-DHAP regimen

Rituximab (375 mg/m^2^) was administered i.v. on day 1 using standard supportive therapy. Dexamethasone (40 mg) was given orally for 4 consecutive days. Cytarabine (2 g/m^2^) was administered on day 2 as a 3-h infusion and was repeated after 12 h. Cisplatin (25 mg/m^2^) was applied on days 1 to 4 as a 3-h infusion. Dose modifications were implemented according to renal function as follows: At a glomerular filtration rate (GFR) below 60 ml/min/1.73 m^2^, cytarabine was reduced to 75 % of the original dose, and to 50 % at a GFR below 50 ml/min/1.73 m^2.^ Cisplatin was reduced to 60 % and 50 %, respectively. A GFR below 30 ml/min/1.73 m^2^ was considered a contraindication for cytarabine und cisplatin. Cycles were repeated every three weeks. For prevention of chemotherapy-induced nausea and vomiting, intensified oral supportive medication (dexamethasone 8 mg and granisetron hydrochloride 2 mg days 1 to 4, aprepitant 125 mg day 1, aprepitant 80 mg day 1 to 6) was used [9].

### Assessment of clinical responses

Response to R-DHAP was evaluated by clinical examination and computed tomography scan of the involved lymph node regions according to standardized response criteria for non-Hodgkin lymphomas [10].

### Assessment of renal toxicity

Renal toxicity was assessed by measuring serum creatinine levels up to three days before each R-DHAP cycle and two to three weeks after termination of each R-DHAP cycle. GFR was calculated according to the Chronic Kidney Disease Epidemiology Collaboration (CKD-EPI) equation [11]. GFR above 90 ml/min/1.73 m^2^ was considered normal renal function. GFRs of 89-60, 59-30, 29-15 and <15 ml/min/1.73m^2^were graded as stage II, III, IV or V renal function impairment.

### Statistical analysis

**Fig. 1:**
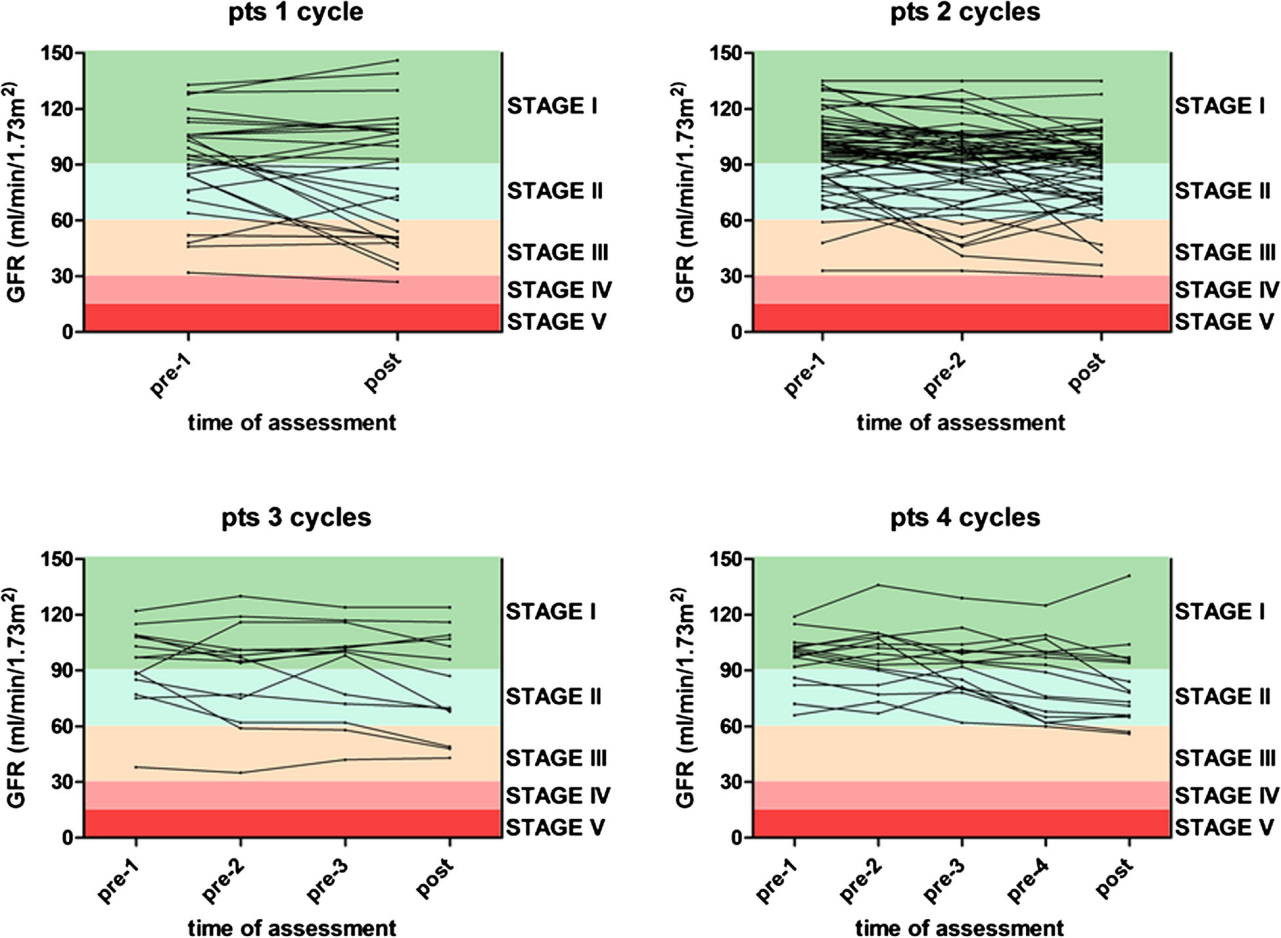
GFR in dependency of the number of R-DHAP courses. The diagrams show the GFR separately for patients who underwent one (**a**, *n* = 31), two (**b**, *n* = 61), three (**c**, *n* = 14) or four (**d**, *n* = 16) courses of R-DHAP, respectively. The data are presented as minimum, first quartile, median, third quartile and maximum

Patients were grouped according to the overall number of received R-DHAP courses (minimum one, maximum four). Descriptive statistics and comparison between the groups was performed by Prism 5.03. Data are given as absolute numbers and percentage, and if not otherwise stated as mean and standard deviation (SD). To increase visual evidence and to allow better comparison, GFR levels are described as mean and SD in the text and illustrated as box plots (minimum, first quartile, median, third quartile and maximum, Fig. 1 and 3). For comparison of categorical variables (gender, stage at first diagnosis, previous therapy, time to relapse after last treatment and response to R-DHAP) Freeman-Halton extension of Fisher's Exact test was used. To identify differences among groups’ means (age at first diagnosis, age at first R-DHAP and duration between last treatment and first R-DHAP) an analysis of variance was performed. Mean differences in individual pairwise comparisons of pre- and post-treatment GFR values were made using paired two tailed student's t test. A *P* < 0.05 was considered statistically significant.

## Results

### Patient characteristics

A total of 122 patients with relapsed or refractory DLBCL were treated with R-DHAP as second-line therapy at our institution. The mean age at first R-DHAP was 57 (SD 12) years, and the male:female ratio was 2:1. 113 (93 %) patients had previously received CHOP-like treatment. In 96 (79 %) patients, first line therapy included rituximab. Most patients had progressive disease or relapsed within 12 months after first treatment. Detailed patient characteristics and treatment details are summarized in Table 1.

**Table 1:** Patient characteristics and response to R-DHAP

	Overall cohort	1 cycle	2 cycles	3 cycles	4 cycles	*P*-values
		R-DHAP	R-DHAP	R-DHAP	R-DHAP	
Patients, n (%)	122 (100)	31 (25)	61 (50)	14 (12)	16 (13)	/
Gender, n (%)						0.17
Male	82 (67)	20 (65)	37 (61)	11 (79)	14 (88)	
Female	40 (33)	11 (35)	24 (39)	3 (21)	2 (12)	
First diagnosis and previous Tx						
Mean age at first diagnosis (SD)	53 (12)	53 (13)	54 (11)	51 (13)	54 (12)	0.73
Stage at first diagnosis, n (%)						**0.01**
I	7 (6)	1 (3)	2 (3)	4 (29)	0 (0)	
II	26 (21)	4 (13)	14 (23)	3 (21)	5 (31)	
III	39 (32)	12 (39)	19 (31)	3 (21)	5 (31)	
IV	48 (39)	13 (42)	26 (43)	3 (21)	6 (38)	
Not known	2 (2)	1 (3)	0 (0)	1 (7)	0 (0)	
CHOP-like therapy, n (%)	113 (93)	29 (94)	58 (95)	12 (86)	14 (88)	0.35
Other, n (%)	8 (6)	2 (6)	2 (3)	2 (14)	2 (12)	
Not known, n (%)	1 (1)	0 (0)	1 (2)	0 (0)	0 (0)	
Rituximab-containing, n (%)	96 (79)	30 (97)	47 (77)	10 (71)	9 (56)	**0.01**
Relapse						
TTR after last treatment, n (%)						**0.01**
Refractory or <12 months	79 (65)	26 (84)	40 (66)	6 (43)	7 (44)	
≥12 months	43 (35)	5 (16)	21 (34)	8 (57)	9 (56)	
Mean age at 1. R-DHAP (SD)	57 (12)	56 (15)	57 (11)	56 (13)	57 (13)	0.98
Months between last treatment and 1. R-DHAP, mean (SD)	19 (29)	8 (15)	17 (28)	43 (47)	27 (23)	**<0.01**
Response to R-DHAP, n (%)						**<0.01**
CR	19 (16)	0 (0)	11 (18)	5 (36)	3 (19)	
CRu	6 (5)	0 (0)	5 (8)	0 (0)	1 (6)	
PR	39 (32)	3 (10)	18 (30)	6 (43)	12 (75)	
StD	18 (15)	5 (16)	11 (18)	2 (14)	0 (0)	
PD	35 (29)	18 (58)	16 (26)	1 (7)	0 (0)	
Not known	5 (4)	5 (16)	0 (0)	0 (0)	0 (0)	

*CHOP* cyclophosphamide, hydroxydaunorubicin, vincristine, prednisone, *CR(u)* complete response (unconfirmed), *PBSC* peripheral blood stem cell collection, *PD* progressive disease, *PR* partial remission, *R-DHAP* rituximab, dexamethasone, cytarabine, cisplatin, *StD* stable disease, *SD* standard deviation, *TTR* time to relapse. Statistically significant *P*-values are indicated in bold.

### Exposure and response to R-DHAP treatment

Overall, 256 R-DHAP cycles were administered. 31 (25 %), 61 (50 %), 14 (12 %) and 16 (13 %) patients received one, two, three or four R-DHAP courses, respectively. Dose adjustments were applied in only 5 (4 %) patients who in total received 11 R-DHAP courses. In one patient cytarabine was reduced to 50 % due to advanced age (>70 years) and poor general condition. One patient received carboplatin instead of cisplatin due to stage III renal impairment. In two patients, both cytarabine and cisplatin dose adjustments were necessary due to a GFR <50 ml/min/1.73 m^2^. In one patient, reasons for and extent of R-DHAP dose adjustments were not documented. In 5 patients the response to R-DHAP treatment was not available. 19 (16 %), 6 (5 %), 39 (32 %) and 18 (15 %) patients achieved CR, CRu (complete response unconfirmed), partial remission (PR) and stable disease (StD), respectively. 35 (29 %) patients had progressive disease (PD), which predominantly occurred after the first two cycles of R-DHAP (Table 1).

### Renal toxicity

**Fig. 2:**
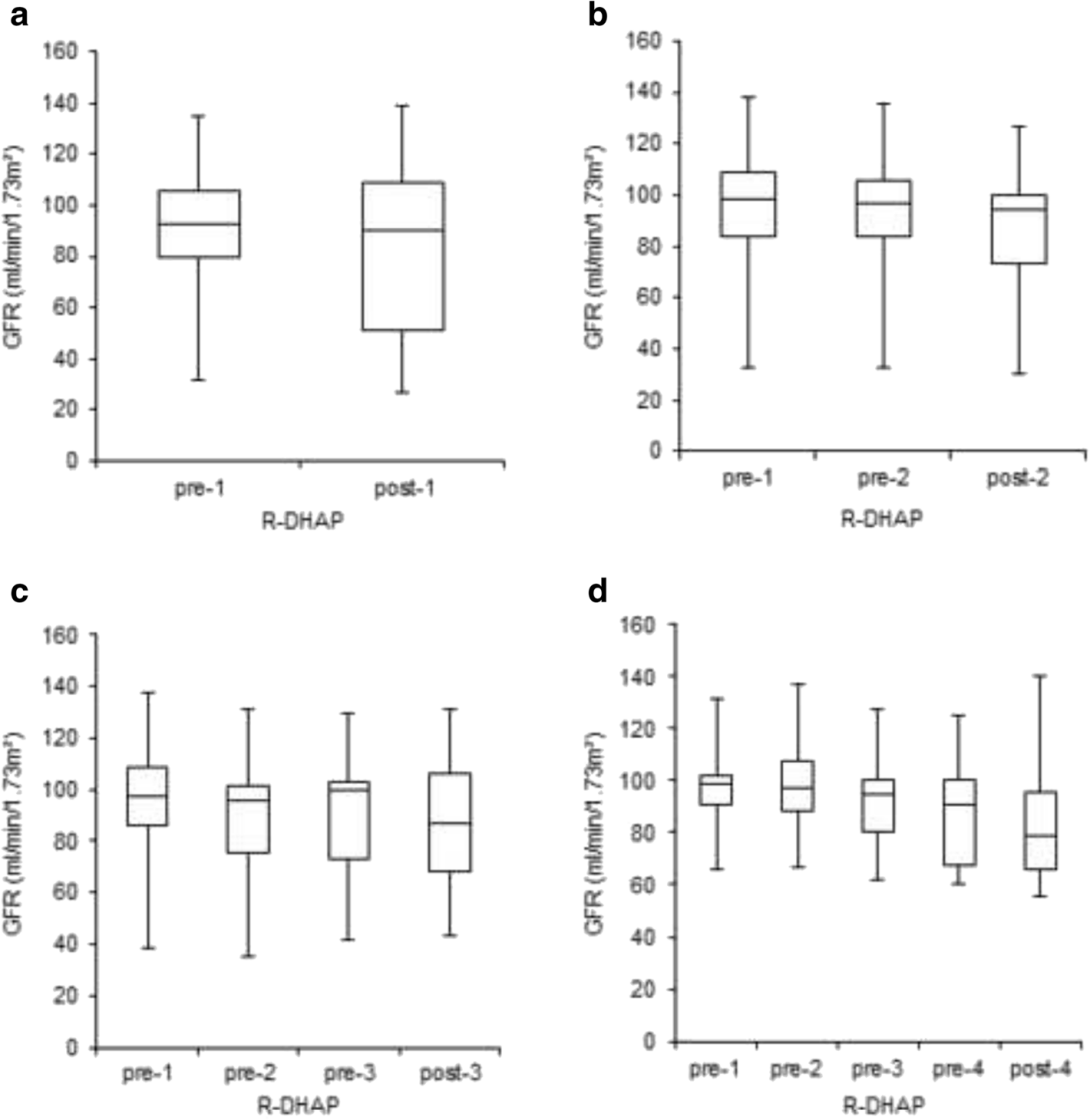
Renal function during R-DHAP treatment by stage. GFR and corresponding renal function by stage during the course of therapy is shown separately for every single patient who underwent one (**a**, *n* = 31), two (**b**, *n* = 61), three (**c**, *n* = 14) or four (**d**, *n* = 16) cycles of R-DHAP

In patients who received one cycle of R-DHAP the initial mean GFR was 91 (SD 25) ml/min/1.73 m^2^. 17 (55 %) patients had normal renal function. Ten (32 %) and four (13 %) patients showed an initial stage II and III renal impairment. After one course of R-DHAP, the GFR remained stable (mean 84, SD 34 ml/min/1.73 m^2^, *P* = 0.06, Fig. 1a). We detected individual deterioration in renal function by one toxicity stage in nine patients and by two toxicity stages in two patients (Fig. 2a).

In patients who received two courses of R-DHAP, the initial mean GFR significantly decreased from 97 (SD 20) ml/min/1.73 m^2^ to 92 (SD 22) ml/min/1.73 m^2^ after the first chemotherapy cycle (*P* < 0.01) and to 88 (SD 20) ml/min/1.73 m^2^ after the second course of R-DHAP (*P* < 0.001, Fig. 1b). Before treatment, 42 (69 %) patients had normal renal function. Eight of these patients had worsening kidney function by one stage and one by two stages. In all but one of 15 (25 %) patients who showed stage II renal impairment before R-DHAP kidney function remained stable or even improved after two courses of therapy. In three (5 %) patients who had stage III renal function impairment before therapy, no decrease in renal function was observed (Fig. 2b). In one patient initial renal function was not available.

A significant GFR decrease in patients who have been treated with three R-DHAP courses from initially 94 (SD 21) to 84 (SD 27) ml/min/1.73 m^2^ after the third course of therapy (*P* = 0.04) was observed. While the GFR was almost stable over the first two cycles of chemotherapy the main decline occurred after the third R-DHAP cycle, where the mean GFR fell by 7 ml/min/1.73 m^2^ (Fig. 1c). We noticed a worsening in renal function from stage I to stage II in three patients and from stage II to stage III in two patient of this subgroup (Fig. 2c).

Similarly, in patients who received four R-DHAP cycles, the initial mean GFR of 96 (SD 14) ml/min/1.73 m^2^ remained stable over the first two cycles. After the third and fourth course of R-DHAP, the mean GFR significantly fell to 87 (SD 20, *P* = 0.01) and 83 (SD 22, *P* < 0.01) ml/min/1.73 m^2^ (Fig. 1d). In this subgroup we found a decrease in renal function from stage I to stage II in six patients and from stage II to stage III in two patients (Fig. 2d).

**Fig. 3:**
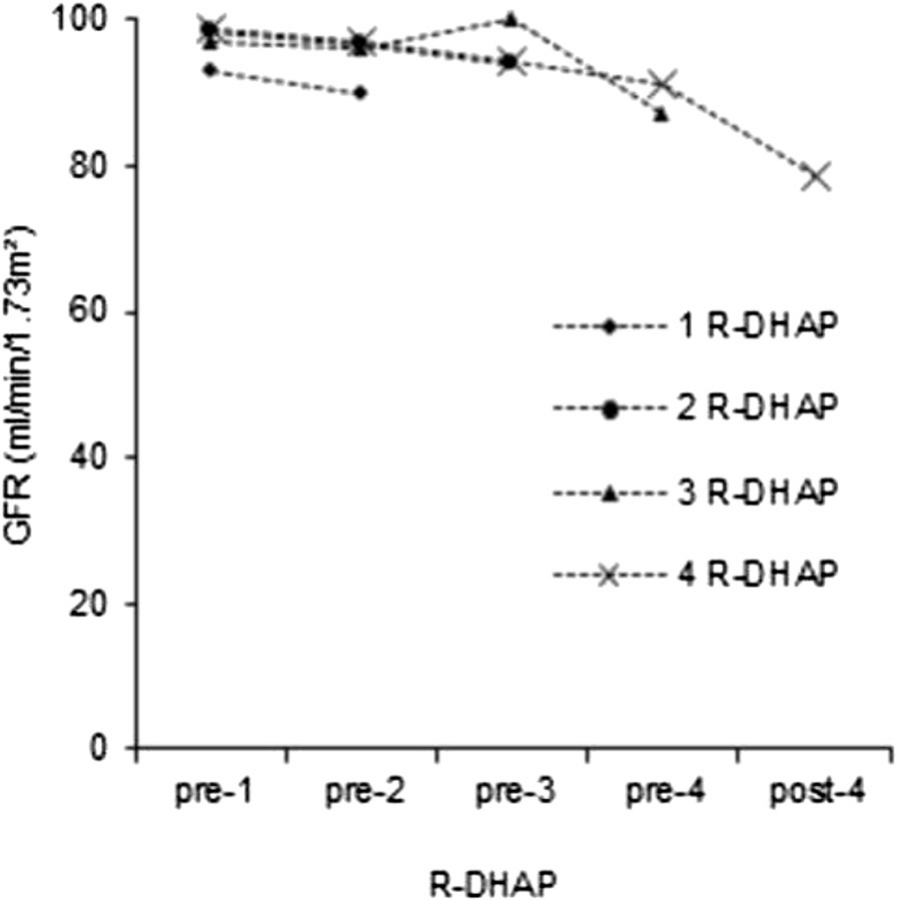
Comparison of GFR between the subgroups. The diagram summarizes the development of median GFR in the course of therapy subdivided by the number of R-DHAP cycles received

Figure 3 summarizes the GFR separated by subgroup in dependency of the course of R-DHAP therapy.

## Discussion

We here performed an analysis of 122 patients with relapsed or refractory DLBCL treated with a modified R-DHAP regimen. Contrasting the practice of other centers, the total dose of cisplatin was maintained but spread over 4 consecutive days to minimize renal toxicity. Our aim was to assess renal toxicity associated with this regimen using a “real-life” population not selected for clinical trials. This series was homogeneous according to the diagnosis relapsed or refractory DLBCL, gender distribution, previous CHOP-like therapy and age at first R-DHAP but heterogeneous according to stage at first diagnosis, time to elapse after last treatment and duration between last treatment and first R-DHAP among the four R-DHAP groups.

We show that the modified R-DHAP regimen as administered in our institution is efficient and safe in this patient group. We observed chemosensitivity in 68 % of patients, and PD occurred in only one third of the patients. This is in accordance with response rates reported for conventional R-DHAP regimens applied in prospective trials, where response to therapy was observed in 76 % of the patients (7).

Importantly, modified R-DHAP did not lead to severe renal impairment in our cohort. When analyzing GFR according to the number of cycles received, renal impairment stage III was observed in less than 10 % and stage IV only in less than 1 % of patients, and none of the subgroups had an initial average GFR of less than 60 ml/min/1.73 m^2^. Within subgroups we found an additive renal toxicity over the course of R-DHAP treatment, but a statistically significant GFR decrease was observed only after the third and fourth R-DHAP course. A direct prospective comparison between modified and conventional R-DHAP regimens has not been conducted yet and unfortunately, comparisons of retrospective results published by different centers on non-modified R-DHAP are hampered by the heterogeneity of patient cohorts and treatment approaches [7, 8, 12, 13].

In particular, renal toxicity was not assessed in detail and in relation to the number of treatment cycles received the majority of published studies using conventional R-DHAP. In a randomized phase III trial in relapsed or refractory DLBCL comparing the salvage regimes R-DHAP versus R-ICE (ifosfamide, carboplatin, etoposide), stage IV renal toxicity was observed in 11 of 194 patients (6 %) treated with conventional R-DHAP over the course of three cycles (7). In contrast, we observed no stage IV renal toxicity in patients who received three or even four courses of modified R-DHAP.

To the best of our knowledge this is the first detailed evaluation of renal function during R-DHAP treatment in a large series of relapsed or refractory DLBLC patients. Our data show that the administration of 25 mg/m^2^ cisplatin for 4 consecutive days in a 3 h infusion as a component of the R-DHAP regimen is efficient in relapsed or refractory DLBCL and leads to a tolerable additive renal toxicity over up to four R-DHAP courses. Furthermore, this study demonstrates that a detailed analysis of renal function impairment after each chemotherapy cycle is required to assure comparability between different chemotherapy protocols.

## Conclusion

R-DHAP (rituximab, cisplatin, cytarabine, dexamethasone) is an accepted salvage regimen for relapsed or refractory diffuse large B-cell lymphoma patients. Assuming that a lower single dose of cisplatin over several days would reduce renal toxicity, the total dose of cisplatin (100 mg/m^2^) was spread over 4 consecutive days. Renal impairment stage III was seen in 10 % and stage IV only in 1 % of patients indicating minimal renal toxicity of the modified R-DHAP regimen.

## Abbreviations

ASCT: autologous stem cell transplantation
CKD-EPI: Chronic Kidney Disease Epidemiology Collaboration
CHOP: cyclophosphamide, hydroxydaunorubicin, vincristine, prednisone
CR(u): complete response (unconfirmed)
DHAP: dexamethasone, cytarabine, cisplatin
DLBCL: diffuse large B-cell lymphoma
GFR: glomerular filtration rate
HD: high-dose
i.v.: intravenous
PBSC: peripheral blood stem cell collection
PD: progressive disease
PR: partial remission
R: Rituximab
SD: standard deviation
StD: stable disease
TTR: time to relapse
